# Methodological Considerations for Furthering the Understanding of Constraints in Applied Sports

**DOI:** 10.1186/s40798-021-00313-x

**Published:** 2021-04-01

**Authors:** Peter Browne, Alice J. Sweeting, Carl T. Woods, Sam Robertson

**Affiliations:** 1 Institute for Health and Sport (iHeS), Victoria University, Footscray, Melbourne, Victoria Australia; 2 Western Bulldogs Football Club, Footscray, Melbourne, Australia

**Keywords:** Interdisciplinarity, Sports technology, Analytics, Perceptual science, Ecological dynamics

## Abstract

Commonly classified as individual, task or environmental, constraints are boundaries which shape the emergence of functional movement solutions. In applied sport, an ongoing challenge is to improve the measurement, analysis and understanding of constraints to key stakeholders. Methodological considerations for furthering these pursuits should be centred around an interdisciplinary approach. This integration of methodology and knowledge from different disciplines also encourages the sharing of encompassing principles, concepts, methods and data to generate new solutions to existing problems. This narrative review discusses how a number of rapidly developing fields are positioned to help guide, support and progress an understanding of sport through constraints. It specifically focuses on examples from the fields of technology, analytics and perceptual science. It discusses how technology is generating large quantities of data which can improve our understanding of how constraints shape the movement solutions of performers in training and competition environments. Analytics can facilitate new insights from numerous and complex data through enhanced non-linear and multivariate analysis techniques. The role of the perceptual sciences is discussed with respect to generating outputs from analytics that are more interpretable for the end-user. Together, these three fields of technology, analytics and perceptual science may enable a more comprehensive understanding of constraints in sports performance.

## Key Points

- The fields of technology, analytics and the perceptual sciences offer opportunities to further the understanding of constraints in applied sport.
- Developments in the fields of technology, analytics and the perceptual sciences can accelerate the benefits of an interdisciplinary approach to some of sport’s most pervasive performance questions and challenges.

## Introduction

Sport science, in general, has long been criticised for its insular nature, with various sub-disciplines typically looking to solve existing problems internally [1–5]. In research, this has manifested in the establishment and reproduction of sub-discipline-specific methodologies [6, 7]. In practice, this is often observed in the separation of departments (e.g. strength and conditioning, medical and performance analysis) in high-level sporting organisations, culminating in isolated and siloed thinking [8, 9]. These issues may be due to a variety of reasons, such as disciplines researching sport at varying levels from molecular to the environment, whilst also applying discipline-specific terminology [1, 7]. Within the tertiary education sector, the fast growth of sport science has led to a focus on specialisation [10, 11], which has partially been attributed to the lack of an overarching, unifying framework [7, 11]. Furthermore, current practices are often seen to offer the illusion of integration, but do not fully combine methods and techniques alongside theories and concepts [1].

Accordingly, there have been numerous calls for sport science to progress beyond this insularity and embrace an inter- and even transdisciplinary approach [2, 5, 7, 12–15]. Adoption of an interdisciplinary approach in sport, whilst challenging, could serve to (i) coordinate and unify activity, (ii) communicate translatable ideas coherently and (iii) design and quantify activities which support the emergence of complex and adaptive behaviours [1, 7, 8, 14]. For instance, if practitioners could operate more collaboratively, it could improve the allocation of time and resources by limiting the duplication of data collection and analysis. Data and its subsequent analysis could also be better communicated through consistent language which may aid the transfer of concepts and ideas between disciplines [8]. Through this, an enhanced ability to address some of sport’s most pervasive performance questions and challenges could be gained.

A true interdisciplinary approach would see sports performance disciplines working collaboratively to fully encompass principles, concepts, data and methods to solve problems and support practice [7]. This could result in enhancements of learning, which could then be shared between a range of operational areas, like talent identification, talent selection, performance analysis and coaching [14, 16]. Independent methodologies and measurement techniques could be reconciled to build upon and learn from one another. Interdisciplinarity offers collaborative problem-solving which may potentially lead to enhanced inquisition, the identification of new questions and the resolving of existing problems [10]. For interdisciplinarity to occur, new methods and procedures are required, which may challenge engrained and culturally pervasive disciplinary norms.

Frameworks such as ecological dynamics or Newell’s constraint model offer a basis upon which sports performance can be measured [7, 17]. Either has the ability to act as a vehicle upon which an interdisciplinary approach could be implemented [18], and may aid the alignment of methods and data [7]. Ecological dynamics is the integration of concepts from ecological psychology [19], complexity sciences [20] and coordination dynamics [17, 21]. Newell’s constraint model [17] and its application views skill, learning, development and expertise as emergent properties of a functionally adaptable and evolving relationship formed between an individual and the constraints of their environment [22]. It is noteworthy that these rationales are not localised to a single sport science discipline; rather, they seek to enhance the understanding of related concepts such as skill, performance, learning and expertise [23].

Constraints are understood as the boundaries which shape the emergence of functional movement solutions [24] and are commonly classified into individual, task and environmental categories [17]. Individual constraints can be defined as structural (e.g. body dimensions, technical attributes), historical (e.g. development of resilience, experience) and/or functional (e.g. motivation, cognition) [24–26]. Task constraints are typically defined as rules (e.g. laws of the game, boundary markings), task goals and/or instructional features (e.g. coach instruction or umpire feedback) [25, 27–29]. Environmental constraints can be physical (e.g. weather, light, gravity) or sociocultural (e.g. values, cultural beliefs, peer support) [24, 26, 30]. It has been proposed that task constraints are emergent properties of a system which are able to be distributed between the individual and environment [31]. Moreover, constraints have been hypothesised to interact and be correlated via circular causality and can be nested based on characteristic time-scales [31].

An understanding of the manipulation of constraints and their impact on skilled performance is, therefore, central to the design of activities intended to promote performance and learning in sport. This can be achieved through the manipulation of constraints to design representative practice tasks which preserve key information-movement couplings experienced during competition. However, a central feature of constraints and their impact on emergent movement solutions relates to their interaction [23, 24]. The interaction between constraints is often misunderstood in both practice and research given previous methodological limitations relating to their measurement and interpretation [7]. A methodological limitation is the collection of discrete events without accounting for constraints or the context influencing these events [32]. For example, how do constraints such as time in possession, pressure type, playing at home or away and/or fatigue state interact to influence the emergence of skilled actions in team sports [32]? Future research could overcome this through the use of technology to capture constraints like these and then applying a multivariate analysis technique, could help practitioners understand their influence on emergent behaviour.

Fortunately, for multiple reasons, an interdisciplinary approach to measuring constraints in applied sport is arguably more feasible now than ever before. Recent improvements in a number of seemingly disparate fields and disciplines have the potential to progress this opportunity. Using examples from technology, analytics and the perceptual sciences, this review details how advancements in a range of fields can be leveraged to achieve interdisciplinarity and disciplinary integration in high-performance sport.

## Technology

Ongoing, and recently accelerated, improvements in the field of technology have enhanced the measurement of almost all aspects of sport [33]. Sport science disciplines, including coaching and performance analysis, have traditionally used largely manual methods to measure constraints in practice and competition. However, technology has made it possible to capture these constraints more efficiently and accurately as well as in a more detailed manner [7]. These improvements have impacted a range of disciplines, leading to the manufacturing of better-quality hardware, increased feasibility of athlete tracking and enabling the automated capture of events through computer vision. For instance, developments such as video annotation software enabled practitioners to move from pencil, paper and stopwatch techniques to facilitating the recording of match events and corresponding contextual information in greater detail [34]. More recent technological developments have enabled the capture of an athlete’s location on a playing field through global and local positioning systems as well as optical technologies [35, 36]. Presently, such systems are now capable of providing semi- or automated detection of athlete actions [37, 38].

In addition to incremental improvements, some technological developments have facilitated the identification and measurement of variables and metrics which were previously unrecognised in research and practice. For example, eye-tracking, the detection of emotion in competition [39, 40] and automatic marker-tracking systems [36, 37] have offered insight into the non-linear and complex interaction between variables in near real-time—clarity which is not possible with the human eye alone [41, 42]. Consequently, the number of individual constraints which can be recorded has continued to grow with the development and implementation of technology. A selection of these constraints is reported in Table 1. As technology continues to develop, so too will the opportunities to improve the quality of constraint measurements. Further opportunities exist to develop these technologies with knowledge from other disciplines such as agriculture, city planning and the military (Table 1). However, as observations are embedded in context, the measurement and collection of reliable constraint data is required to take place without losing the validity required for scientific rigour and thereby aid in promoting experimental representative design [107].

**Table 1:** A selection of constraints and contextual factors from team sports unless otherwise specified, which can currently be measured, or could be better measured through improvements in technology

Group	Constraint category	Constraint/context	Constraint examples in the literature	How technology can improve measurement of this constraint	Application of technology in other disciplines
Match events	Task	Location and type of match event	Kempton et al. [43], O’Shaughnessy [44]	Automated ball tracking through computer vision	
	Task	Sport specific events, e.g. Australian football—kick type (drop punt, snap, etc.); hockey—hit type	Slade [45], Hughes and Franks [46]	Automated detection of events via computer vision or device on athlete/equipment (i.e. ball or stick)	Traffic event detection [47]
	Task	Shot location - Angle/distance of goal face visible	Pocock et al. [48], Goldsberry [49]	Player and ball tracking aligned with game logs	
	Task	Time in possession - Individual possession length - Length of possession chain - Team split of previous 10 mins	Higham et al. [50], Robertson et al. [32]	Player and ball tracking aligned with game logs	
	Task/individual	Shot trends: ‘hot hand fallacy’ - Team - Individual	Skinner [51], Bar-Eli et al. [52]	Player and ball tracking aligned with game logs	
	Individual	Disposal efficiency - In game - History	Pocock et al. [48], Reich et al. [53]	Player and ball tracking aligned with game logs paired with analytics	
	Task	Available space - Physical pressure - No. of players between ball and goal - Ratio of attackers to defenders	Rein et al. [54], Alexander et al. [55]	Player and ball tracking paired with improved analytics Proximity sensor	Emotional response in crowds [56]
	Task	Kick distance	Blair et al. [57, 58]	Ball tracking Automated measurement through computer vision	Automated detection of distances in cars [59]
	Individual/task	Physical output - Game time played - Time between efforts - High speed metres	Almonroeder et al. [60], Sarmento et al. [61]	Player and ball tracking paired with match events	
	Task	Ball weight	Nimmins et al. [62], Fitzpatrick et al. [63]	Computer vision	Computer vision to estimate weight of livestock [64]
Individual	Task	Coaching - Technique/feedback - Game style - Enable self-regulation	Wulf and Lewthwaite [65], Wrisberg [66]	Recording and natural language processing Speech to text software	Military detection of keywords [67]
	Individual	Dominant side, e.g. preferred foot	Cust et al. [68], Ball [69]	Automated detection through computer vision	
	Individual	Heart function (heart rate, oxygen saturation)	Klusemann et al. [70], Dong [71]	Sensors in uniforms	Sensors built into clothing [72]
	Individual	Mental components, e.g. mental fatigue, brain activity levels, motivation, resilience, confidence, decision-making skill, emotional state	Russell et al. [73], Joshi et al. [39]	Portable brain electrical activity machines	Health sector development of portable EEG (electroencephalogram) [74]
	Individual	Player characteristics, i.e. physical characteristics, experience, playing position	Piette et al. [75], Sarmento et al. [61]		
	Environment	Social • Cultural • Interactions	Anshel et al. [76], Davids et al. [77]	Proximity sensors	Social proximity using Bluetooth [78]
	Individual	Recovery (training load)	Halson [79], Gastin et al. [80]	Ubiquitous monitoring through 24/7 sensors	Health sector monitoring at-risk patients [81]
	Individual	Sleep	Juliff et al. [82], Halson and Juliff [83]	Improvements in sleep tracking technology	Validation of non-invasive sleep technology [84]
Match context	Task	Difference in team quality	Robertson and Joyce [85], Franks et al. [86]		
	Task	Defensive style/intent	Tan et al. [87], Wang et al. [88]	Player and ball tracking aligned with match log	
	Environment	Opposition characteristics, i.e. physical characteristics, experience, playing position	Franks et al. [86], Milanese et al. [89]		
	Task	Scoreboard - Margin/scoring trends	Goldman and Rao [90], Pocock et al. [48]	Automation through computation	
	Environment	Time in season, fixture type	Dellal et al. [91], Soroka and Lago-Peñas [92]		
	Environment	Playing Surface - Material, i.e. grass (hard, soft, wet, dry)	Bartlett et al. [93], Crowther et al. [94]	Racetrack penetrometer Clegg-Hammer Magnetic layer detection	Magnetic layer detection to measure top soil density [95]
	Task	Pitch dimensions, i.e. area, depth of pockets	Klusemann et al. [70], Kelly and Drust [96]		
	Environment	Weather (rainfall, wind, sun position)	Thornes [97], Ely et al. [98]	Wireless sensor network	Weather impact on air traffic management [99]
	Environment	Venue—crowd, stadium type (roof, open), distance travelled, noise	Gama et al. [100], Goldman and Rao [90]	Computer vision to monitor crowd emotion	Emotion tracking for city planning [101]
	Task	Time: elapsed/remaining in - Period - Game	Pettigrew [102], Sandholtz and Bornn [103]	Automation through computational timing	
	Task	Time elapsed since last - Foul - Stoppage - Turnover - Score	Andrienko et al. [104], Skinner [51]	Automation through computational timing	
	Task	Team synergy	Araújo and Davids [105], Araújo et al. [106]	Ball and player tracking aligned with match log Facial expression extraction	Emotion tracking for city planning [101]

To represent the influence of technology on the measurement of constraints, the concept of pressure in a team sport context presents a useful example. As a scientific construct, pressure has been measured in multiple ways; through the proximity of opponents on the field as measured by player tracking systems [104, 108], an athlete’s physiological and emotional response measured via sensors [39, 56] or the context of a game via the scoreboard or time remaining [48, 90]. Adding more data types to define pressure more comprehensively will likely lead to a greater understanding of its influence on performance outcomes.

Extending these ideas, technology can enable greater clarity with regards to the measurement of constraints players experience during competition. Beyond helping practitioners contextualise actions observed during competition, it can assist with the design of practice tasks that are more representative of the requirements of competition to support athlete development and learning [109]. For example, by understanding the key constraints that shape athlete behaviour, practitioners could design them into practice tasks, thereby preserving information-movement couplings to support athletes in becoming more self-regulating in performance [110].

Of course, both researchers and practitioners will always experience some limitations with respect to the volume of data they collect. Furthermore, the continual addition of new types of data has the potential to overcomplicate modelling and limit user interpretability [111]. From a resourcing perspective, it may not be feasible to collect all possible data sources in training or competition environments. In some cases, the feasibility of measurement may be influenced by the sport itself. For example, whilst an inertial movement unit could provide insight into constraints and contextual factors surrounding limb motion, most governing sports organisations restrict the use of such devices during competition [112]. Additionally, ball tracking systems in team sports are becoming more commonplace in practice, yet the resources to analyse and interpret the outputs remain intensive [109, 113]. Thus, finding a feasible ‘sweet spot’ for the collection of data is required in practice to enable the most impactful implementation of technology.

## Analytics

One criticism leveraged at ecological dynamics points towards its complexity, with research to date being largely conceptual or performed in a laboratory [114]. The measurement of constraints in practice has often been reductionist in approach [107], emphasising either one or two constraints that are measured in isolation [32]. This can provide rigour in relation to the methodological approach; however, it is less representative of the environment being explored [107]. This may result in an overly narrow and potentially even misleading interpretation of sports performance by not accounting for, or misrepresenting, the nuances of a complex system [115–117]. Thus, whilst the literature referred to in Table 1 is encouraging with respect to the large volume of constraints captured in performance settings, it is important to recognise that (i) constraints do not act or interact independently and (ii) not everything can be measured and analysed in research or practice.

Analytics refers to the methods based on the computation of statistics and data [118] and, therefore, considers both computation and algorithms. Machine learning, the application of artificial intelligence that affords a system the ability to autonomously learn through experience or example data, has had increased application in sport [119]. Analytics may aid sport science practices in identifying complex and non-linear patterns within datasets. Machine learning techniques have been used in sport to quantify defensive patterns [37], as well as predict events [48, 120] and match outcomes [120, 121]. Improved analytical methodologies allow for more complex phenomena, such as the interaction of players, to be measured [36, 122]. Accordingly, analytical approaches applied are an important aspect of performance research and decision-making [123–125], supporting the experiential knowledge of professional practitioners. To make analytics applicable to industry settings, practitioners may need enhanced skillsets to use data tools which can analyse and summarise big datasets and/or seek to establish bespoke workbooks that automate preferred data visualisations following data capture. In the time-constrained environment of high-performance sport, analytics may streamline decision-making across multiple departmental areas [36, 122, 126].

A key benefit of analytics when applied to sports performance relates to its flexibility. Specifically, in regard to how different machine learning algorithms can often be used interchangeably on the same dataset [127–129]. This can enable a problem to be viewed through multiple lenses which may be implemented or visualised differently based on user preferences. Returning to the abovementioned example, it has been acknowledged that pressure has been analysed in various ways. For example, weighted densities paired with linear and quadratic functions have been used to understand defensive players’ movements through spatiotemporal data in soccer [104], whereas in basketball, matrix factorisation and regression models have been utilised for the same purpose [86]. The creation and measurement of a pressure metric can be achieved with either discrete or continuous variables. Representation of pressure in a categorical format (i.e. ‘high’ or ‘low’) may make for easier stakeholder comprehension and implementation in the applied setting. Irrespective of the format in which the data are represented, however, more context than solely player movement is required to fully measure pressure. Thus, using player density or pitch control [130, 131], alongside score board margin, time remaining and individual traits of an athlete could offer greater insight into the pressure experienced at any given time.

The collection of more data related to different interacting constraints can ultimately make the analysis of variables more difficult. The principle of parsimony is critical within analytics to strike a balance between feasibility and obtaining a high-level understanding of phenomena of interest. Without enhanced analytical tools, the translation of model outputs which contain a large number of variables into meaningful information remains a challenge [111, 132]. Parsimony relates to achieving a balance between collecting enough variables to sufficiently support an evaluation but not so many that only provide small improvements in understanding [133, 134]. Within sport, parsimony is vital to the uptake of new models and decision support systems, as it can reduce data redundancies and optimise time investments. For example, if a model requires five variables to achieve 80% accuracy on a given problem, the time and resources required to collect an additional ten variables to improve accuracy by 5%, may not outweigh the benefit of a slightly less accurate model. This has useful applications in scenarios whereby a model requires implementation across multiple environments. For instance, comparing junior athlete performance with professional competition for team sport scouting purposes may see the user having access to differing levels of data, leading to a lack of direct comparability of performances. Many variables used within the professional competition may not be available at lower levels; thus, invoking the notion of parsimony forces the user to focus on including those variables that are not only the most important, but also readily available across all levels of competition.

Parsimony also helps to avoid problems with overfitting. Overfitting describes a model, which is generated specifically to a training dataset, but where the results are not generalisable or validated on a new or unseen test dataset [123, 135]. In the example above, a scouting model used in professional competition may show accurate predictive performance when applied to professional players, but due to its large number of inputs (amongst other factors) may generalise less well to other competition levels [124]. Striking a compromise between parsimony and model accuracy is a complicated exercise, particularly in the field, but is an increasingly important consideration as sports performance models become more complex and detailed.

## Perceptual Science

The growth in data along with the enhanced analysis of these data have been emphasised to this point of the review. However, without the output from such analyses being appropriately communicated to and interpreted by key stakeholders, the gains achieved by sport science will go unrealised. Learnings from the perceptual sciences could hold the key to assist in this area. Perceptual sciences refers broadly to the integration of neuroscience, computer science and psychology with the aim to understand the link between external properties and cognition [136, 137]. Specifically, components of cognitive science such as psychophysics, alongside the art of visualisations, may be used to enrich the interpretation of analytics and explain why some visualisations better enable the detection of key information. Together, the concepts of cognitive science and the perceptual sciences can provide a foundation and guide for the utilisation of the science and language of visualisations to maximise comprehension and improve the communication of complex phenomena in sport. In doing so, visualisations may enable the user to identify the relationship between an individual and their environment with enhanced clarity by preserving the complex and non-linear interactions at work, which are typically reduced through traditional, linear approaches.

Visualisations have been proposed as a method to leverage data communication to highlight findings clearly with precision and efficiency. This is required as despite the improvements in technology and analytics, a linear improvement in performance has not occurred [116]. This may be partially due to a user’s reduced ability to gain insight from numerical data, as tables may be inferior to visualisations in communicating results [138]. Visualisations provide a tool to translate numbers into a simpler medium for ease of interpretation and implementation [136]. This may be due to the increased cognitive load required to comprehend numerical data compared with visualisations [136, 139]. Thus, as analytical model outputs become more complex, visualisations can help to support the user’s comprehension. Ultimately, if such output is not interpretable or operationalisable, even the best performing model will not be implemented by key stakeholders in the applied setting [113, 134].

Given the inherent complexity of ecological dynamics, visualisations are critical in their ability to indirectly convey key information. Visualisations are an essential tool to enable the appreciation of complex and multidimensional constraints in a system. The ability to visualise multiple variables may further enhance the communication of complex information. For instance, five dimensions can be displayed and manipulated through the two regular axes as well as hue, shape and size of data points. The impact of visualisations on stakeholder decision-making has been examined in forecasting, communication and planning [140–143]. Furthermore, visualisation aesthetics have been linked with an individual’s engagement, enjoyment and memorability [144–147]. However, an awareness of inherent biases is also required in the generation of visualisations. Biases are cognitions which prejudice decision-making [148]. For instance, as the number of components displayed in a bar chart increases, the accuracy with which the chart is interpreted decreases [149]. The transition from data and analysis to the creation of a visualisation may aid in the uptake of information and thus, insight in the applied setting [136].

**Fig. 1:**
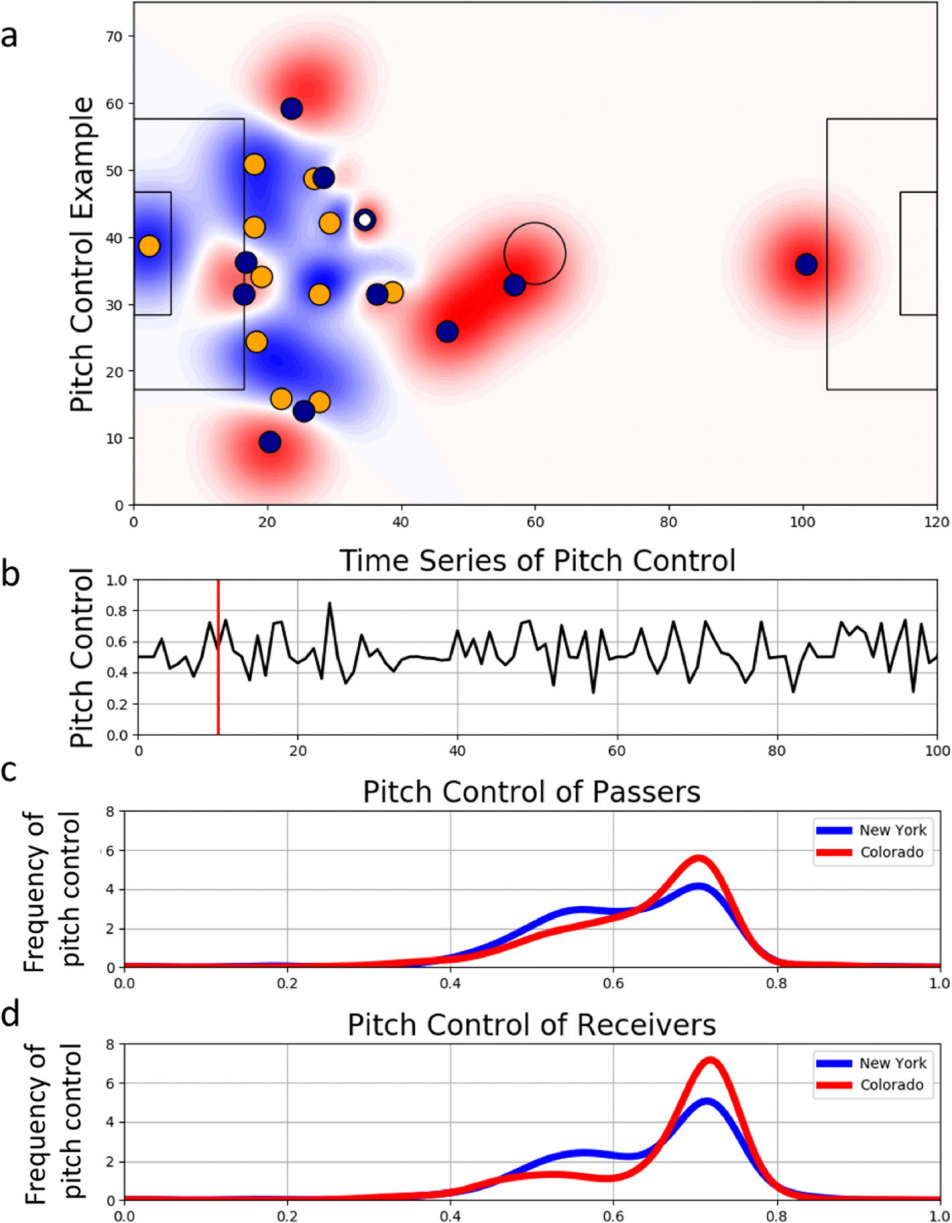
Examples of different ways pressure may be visualised via an exemplar from football. The metric of pitch control is used, a concept which defines the probability that an athlete or team has control of a specific point. **a** Static image of the pitch with player locations and pitch control at the time indicated by the red line in **b**. Ball possession is represented by the white circle. **b** Time series of pitch control of the attacking team calculated as a minute by minute average of pitch control over course of a game, where 1 represents total pitch control by team 1, 0.5 represents equal levels of pitch control of both teams and 0 relates to total pitch control by team 2. The red line indicates the time **a** was taken from. **c** Density plot of the level of pitch control of passer. **d** Density plot of the level of pitch control of receiver

Returning to the pressure example discussed in earlier sections, it is apparent that despite their visual potential, pressure or defensive actions are often reported as aggregate data, for example as a frequency count in a table [110, 150]. However, in scenarios whereby continuous pressure metrics have been proposed [104, 130], visualisations can be used in different ways to provide alternate insights with the same data. For example, Fig. 1 provides an example of how pitch control can be used to visualise pressure. Pitch control is a concept which defines the probability that an athlete or team has control of a specific point of a certain region of the pitch at a given time point [130]. It is based on athlete location, velocity and relative distance from the ball at a given time point, where the aggregate influence of each team’s athletes is calculated on a continuous scale to provide a measure of pitch control [130]. Specifically, Fig. 1a shows an overview of a passer in football (represented by the white dot) at a discrete moment in time during a match. The darker the blue area the more pressure experienced by the passing player, based on their level of ‘pitch control’. Such a visualisation can be used to provide further context to the pressure not just surrounding the passer, but the options available to them. Furthermore, the level of pitch control varies throughout a game, which can be visualised as a time series to display how pressure changes for the team in possession throughout a match (Fig. 1b). Thus, ‘1’ would represent total pitch control by team 1, 0.5 represents equal levels of pitch control by both teams and ‘0’ relates to total pitch control by team 2. Furthermore, visualising pitch control at the location of the passer and receiver may provide insight into game style, risk-taking behaviour and decision-making (Fig. 1c, d). For instance, a team may relinquish some pitch control at the location of the passer to create more space at the location of the receiver. This tactic may increase the pressure, or decrease pitch control, at the ball location but lessen the pressure for the receiver. Thus, visualisations may enable the facilitation of the improvements in technology and analytics to be realised. When operationalised in unison, they may help aid decision-making and encourage interdisciplinarity—demonstrated by this pressure example, which uses player tracking data alongside algorithms to generate a pitch control metric and visualisations to help convey these data in a usable format. Thus, appropriate visualisations using the same data or model can improve the communication of findings to key stakeholders and facilitate rapid interpretation and implementation [139, 151, 152].

## Conclusion

This narrative review has provided some methodological considerations for the measurement of constraints though an interdisciplinary approach. The benefits of an interdisciplinary approach could arise from greater consistency between disciplines, more efficient workflows and optimised communication procedures [8]. These improvements may allow for questions to be answered more completely, rather than solutions that have origins and applications in a single discipline. This narrative review specifically discussed how the continually developing fields of technology, analytics and perceptual sciences are situated to help guide and support sport science to make the integration between disciplines more feasible. Importantly, these fields are not all encompassing and many others exist which can further the measurement of constraints in applied sport. Whilst other fields may further the development of constraint measurement, interdisciplinarity can also be encouraged through applying an overarching framework [7]. By embracing a truly interdisciplinary approach, progress on many of sport science’s most pervasive and important questions can be realised. However, for sport science to continue to progress towards interdisciplinarity, more needs to be done to create environments open to change, where improvements can transcend sub-disciplines. Furthermore, academic institutions need to provide training and education which are supportive of interdisciplinary approaches, as opposed to driving a continued discipline speciality. This may see high-performance sports organisations reassess structures, move away from siloing departments, towards creating integrated, functioning environments where time and resources are available to be utilised in an interactive way. The removal of such barriers may aid sport scientists in adopting the principles of interdisciplinarity.

## Data Availability

Not applicable

## Abbreviations

EEG: Electroencephalogram
